# Evidence for acute contraction-induced myokine secretion by C2C12 myotubes

**DOI:** 10.1371/journal.pone.0206146

**Published:** 2018-10-24

**Authors:** Yasuro Furuichi, Yasuko Manabe, Mayumi Takagi, Miho Aoki, Nobuharu L. Fujii

**Affiliations:** Department of Health Promotion Sciences, Graduate School of Human Health Sciences, Tokyo Metropolitan University, Tokyo, Japan; University of Minnesota Medical Center, UNITED STATES

## Abstract

Skeletal muscle is considered a secretory organ that produces bioactive proteins known as myokines, which are released in response to various stimuli. However, no experimental evidence exists regarding the mechanism by which acute muscle contraction regulates myokine secretion. Here, we present evidence that acute contractions induced myokine secretion from C2C12 myotubes. Changes in the cell culture medium unexpectedly triggered the release of large amounts of proteins from the myotubes, and these proteins obscured the contraction-induced myokine secretion. Once protein release was abolished, the secretion of interleukin-6 (IL-6), the best-known regulatory myokine, increased in response to a 1-hour contraction evoked by electrical stimulation. Using this experimental condition, intracellular calcium flux, rather than the contraction itself, triggered contraction-induced IL-6 secretion. This is the first report to show an evidence for acute contraction-induced myokine secretion by skeletal muscle cells.

## Introduction

Exercise presents a wide variety of health benefits, such as the prevention of type 2 diabetes, enhanced immune function, the prevention of Alzheimer’s disease, and a reduction in the risk of developing cancer [1]. These effects are not limited to skeletal muscles but also extend to many other organs in the body. A highly sought-after goal for research scientists in this area is to uncover the mechanisms underlying the benefits of exercise and to utilize these mechanisms to prevent and treat diseases. Muscle-derived secretory proteins, known as myokines, have recently attracted attention as key players in the crosstalk between skeletal muscle and other organs [2–5]. Decades ago, the levels of some cytokines in circulating blood were first shown to increase during exercise, and these cytokines were believed to be produced by contracting skeletal muscles [6]. Currently, skeletal muscle is considered a secretory organ that produces bioactive proteins that affect distant organs in an endocrine manner and the surrounding tissues in an autocrine and paracrine manner. Several novel myokines have been reported [7], but many undiscovered proteins remain to be characterized since a recent omics analysis has implied that hundreds of proteins are secreted by skeletal muscle cells [8].

One of the major problems in the study of myokines is that their secretion from skeletal muscle cells has not been unequivocally proven. In some reports, increased levels of target proteins in the plasma after acute exercise were used as an index of myokine secretion [9, 10]. However, the possibility that the increase in protein levels in the circulating blood arises from proteins secreted by other cells that surround the muscle tissue, such as vessels, neurons, or blood cells, cannot be neglected. Cell culture experiments have been performed to eliminate contamination with proteins produced by non-muscle cells. The murine-derived C2C12 skeletal muscle cell line is widely used to discover myokines, and several dozen proteins have been detected in conditioned media from C2C12 myotubes [11–15]. Furthermore, we and other groups have succeeded in developing a cultured C2C12 myotube contraction system that enables us to investigate contraction-induced myokine secretion [7, 14].

Interleukin-6 (IL-6) was initially described as a myokine and is thought to be secreted by skeletal muscles following exercise. The plasma IL-6 level dramatically increases (up to 100-fold) during acute exercise and begins to decrease immediately after exercise [16, 17]. This observation prompted the speculation that acute exercise-induced (or muscle contraction-induced) IL-6 secretion from muscle cells might affect various organs through paracrine and endocrine pathways [18]. However, to our knowledge, direct evidence showing that myokine secretion by skeletal muscle cells is enhanced by contraction is not available. Although the level of IL-6 in conditioned medium from C2C12 myotubes increased following electrical stimulation in a few studies, IL-6 secretion required contraction for more than 12 hours, suggesting that constitutive secretion, but not regulatory secretion, is probably upregulated due to the increase in *de novo* synthesis of the IL-6 protein following chronic contraction [14, 19–21]. Researchers have not clearly determined whether IL-6 secretion by skeletal muscles is regulated by acute contraction and, therefore, whether a regulated secretion system is present in skeletal muscle cells. An understanding of the molecular mechanism underlying the secretion of regulatory myokines is required for the production of new drugs, which may induce beneficial myokine secretion in patients unable to perform exercise [22].

Here, we demonstrated that IL-6 is secreted from C2C12 myotubes following a 1-hour acute muscle contraction, evoked by electrical stimulation. Importantly, changing the cell culture medium to serum-free buffer causes the release of large amounts of proteins from C2C12 myotubes, which obscures the contraction-induced myokine secretion. Once protein release was abolished, the secretion of IL-6 from the myotubes increased in response to a 1-hour contraction. Furthermore, using this experimental condition, intracellular calcium flux, rather than the mechanical movement of muscle cells, triggered contraction-induced IL-6 secretion. These findings provide evidence that skeletal muscle cells present a contraction-regulated secretion mechanism for myokines.

## Materials and methods

### Cell culture

C2C12 myoblasts (American Type Culture Collection, Manassas, VA, USA) were seeded into 4-well rectangular plates at a density of 2.0*10^5^ cells/well in 3 mL of growth medium comprising DMEM (25 mM glucose; Invitrogen, Carlsbad, CA, USA), supplemented with 10% fetal bovine serum (Bio West, Nuaillé, France) and 1% penicillin-streptomycin. Plates were maintained in an incubator at 37 °C in a 5% CO_2_ atmosphere. When the cells reached 80% confluence, the medium was switched to differentiation medium, consisting of DMEM supplemented with 2% calf serum (Bio West) and 1% non-essential amino acids (Invitrogen) (day 0). Five days after differentiation, cells were used in the experiment.

### C2C12 myotube contraction and conditioned media extraction

C2C12 myotube contraction was induced using an electrical stimulation system, as previously described [7, 23], with minor modifications. Briefly, the cells were washed twice with 6 mL of PBS and were maintained in serum-free media: DMEM without phenol red supplemented with 4.0 mM l-glutamine or KRB buffer containing 2 mM pyruvate. Four-well plates were connected to the electrical stimulation apparatus, a four-well C-Dish (Ion Optix Corp., Milton, MA, USA), and were stimulated with an electrical pulse generator (Uchida Denshi, Hachioji, Japan). The electrical current in the medium was measured using a current monitor (Uchida Denshi, Hachioji, Japan). Differentiated C2C12 myotubes were stimulated with 1-Hz electric pulses for 1 hour at various voltages and for different pulse durations.

For culture experiments involving the use of inhibitory reagents, *N*-benzyl-*p*-toluene sulfonamide (BTS) and/or EGTA were added to the culture medium at a final concentration of 50 μM and 10 mM, respectively. BTS was dissolved in DMSO, and a medium containing DMSO at a final concentration of 0.05% was used for control cultures.

After contraction, the conditioned medium was collected and centrifuged at 2,000 × g for 15 minutes at 4 °C to remove the residual cells. The sample was centrifuged again at 12,000 × g for 35 minutes at 4 °C. The supernatant was filtered using 0.22 μm membrane filters (Millipore, Watford, UK) and was concentrated using 3 kDa cut-off centrifugal filters (Millipore, Watford, UK).

### Transfection

C2C12 myoblasts were transiently transfected with 2 μg of pCMV IL-6 or pCMV IL-6-HA expression vectors (purchased from Sino Biological Inc., Beijing, China), 187.5 μL of Lipofectamine 2000, and 30 μL of Plus reagent (Invitrogen), according to the manufacturer’s instructions.

### Western blotting

Conditioned media samples were separated by sodium dodecyl sulfate-polyacrylamide gel electrophoresis, and transferred to polyvinylidene fluoride membranes. Membranes were blocked with Tris-buffered saline containing 5% non-fat dry milk and 0.1% Tween 20. Membranes were then incubated overnight with the appropriate primary antibodies (anti-IL-6 (AF406NA, R&D Systems, Minneapolis, MN, USA), anti-β-actin (4967, Cell Signaling Technology, Massachusetts, USA), anti-CD9 (CBL162, Millipore, Watford, UK), anti-IL-15 (MAB447, R&D Systems), anti-SPARC (5420, Cell Signaling Technology), or anti-CXCL5 (ab9983, Abcam)), followed by an incubation with rabbit (GE Healthcare, Buckinghamshire, UK) or goat (Millipore, Watford, UK) secondary antibodies conjugated to horseradish peroxidase. For IL-6, the membrane was blocked with 3% non-fat dry milk, and the antibodies were diluted using IMMUNO SHOT solution (Cosmo Bio, Tokyo, Japan). Blots were subsequently developed using ECL plus (PerkinElmer Life Sciences, Inc. Waltham, MA) and were analyzed with a Luminescent Image Analyzer LAS-4000 mini (GE Healthcare, Piscataway, NJ). Data were quantified using the ImageJ software.

### Statistical analysis

All data are expressed as the mean ± standard error of the mean (SEM). Unpaired *t*-tests were used to compare the data between two groups. Variations per hour were compared using a one-way ANOVA, and a Tukey-Kramer post hoc test was conducted if the ANOVA indicated a significant difference. Differences in IL-6 secretion observed following treatments with inhibitors were assessed using one-way ANOVA followed by Dunnett’s post hoc tests to compare treatment values to DMSO basal control values. The level of significance was set to *p* < 0.05.

## Results

### Changes in IL-6 levels in the cell culture media after the contraction of C2C12 myotubes

**Fig 1:**
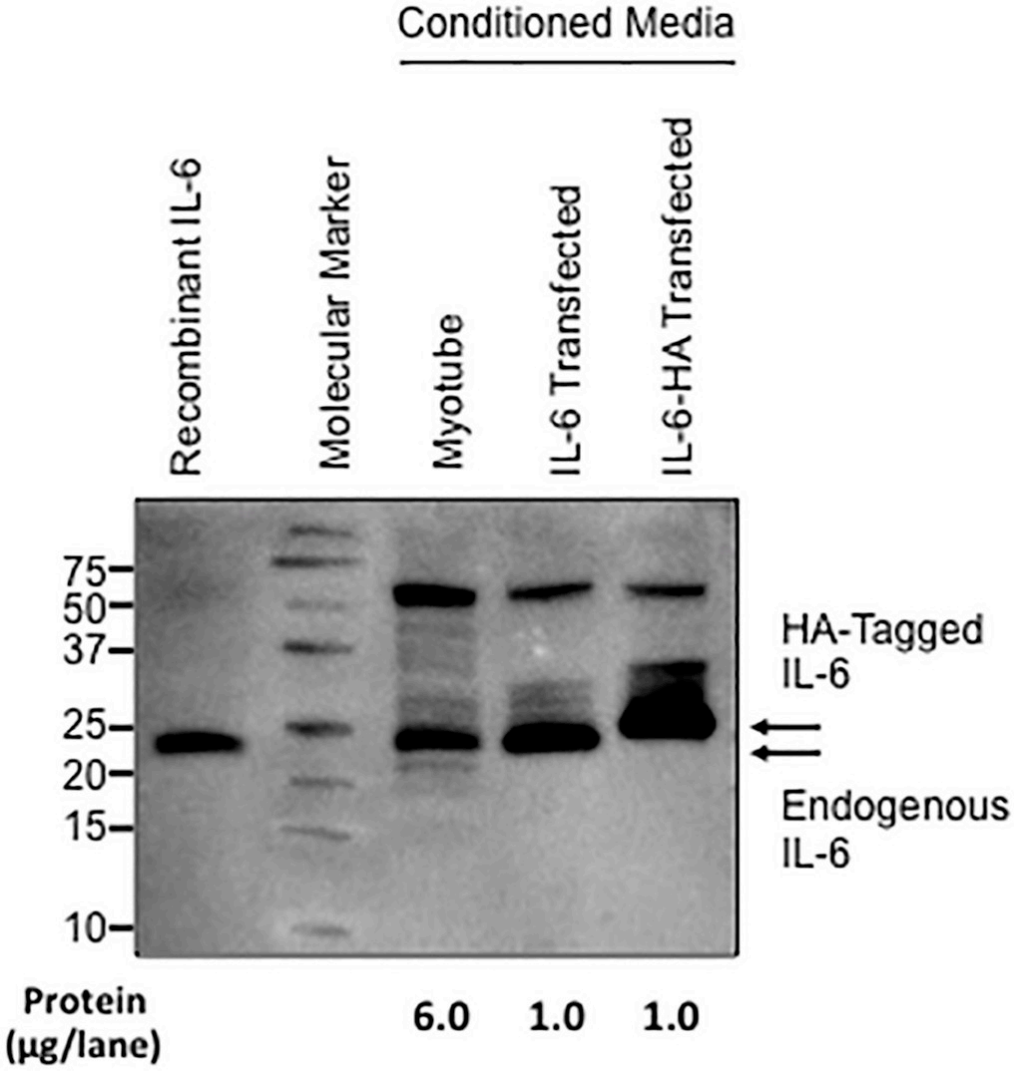
Validation of IL-6 detection using western blotting. A band corresponding to the molecular weight of ~21 kDa was obtained in conditioned media from control (no transfection) and IL-6-transfected C2C12 myotubes. The molecular weight of this band was also identical to recombinant IL-6. The band obtained from the conditioned media from HA-tagged C2C12 myotubes was shifted upwards, as predicted.

We focused on IL-6 to investigate contraction-induced myokine secretion from muscle cells because it is considered an exercise/contraction-stimulated myokine [24]. The concentration of the IL-6 protein in plasma and conditioned media from cultured cells was measured using an enzyme-linked immunosorbent assay (ELISA) kit, as reported in several previous studies [10, 16, 20]. However, the quantification of target proteins using an ELISA is based on the formation of an antigen-antibody complex, which may, in some cases, be affected by interference from non-specific cross-reacting proteins [25]. We chose western blotting, which provides molecular weight information for target proteins and is more accurate in quantifying protein levels to avoid this potential limitation. Expression vectors encoding IL-6 or HA-tagged IL-6 were transfected into C2C12 myoblasts. After differentiation, conditioned media were collected, and assayed using western blotting with a specific antibody against IL-6 (Fig 1). We confirmed the accuracy of IL-6 protein detection comparing by those positive controls. The details are described in the Figure legend.

**Fig 2:**
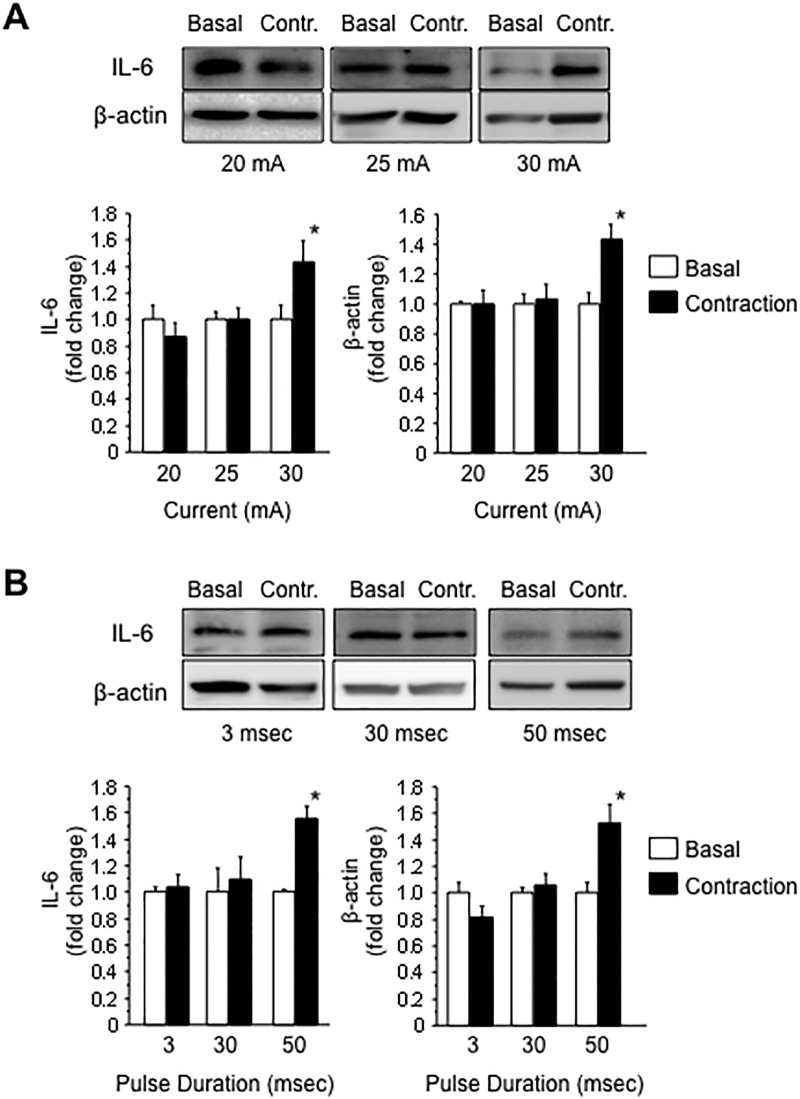
IL-6 secretion by C2C12 myotubes increases in a manner dependent on the intensity of electrical stimulation and is associated with cell damage. Levels of IL-6 and β-actin in the conditioned media from C2C12 myotubes maintained in a basal state and after 1 hour of electrical stimulation were quantified using western blotting. Electrical stimulation of C2C12 myotubes was performed using various currents (A) and pulse durations (B). Values are expressed relative to the basal medium and are presented as the mean ± SEM (n = 6 (a), n = 4 (b)). *: p < 0.05 compared with the basal medium.

We used an electrical stimulation system [7, 23] and contracted C2C12 myotubes in serum-free Dulbecco’s modified Eagle’s medium (DMEM) for 1 h. The IL-6 level in the conditioned media significantly increased after contraction induced by electrical stimulation using a 30-mA current compared to that in the culture media of non-contracted C2C12 myotubes. However, the level of β-actin, a marker of cell damage [26], was also increased in media from contracted myotubes. Therefore, the increase in IL-6 levels was caused by the leakage of intracellular proteins rather than secretion (Fig 2A). We also investi0067ated the effects of a pulse duration during electrical stimulation because extended electrical stimulation potentially induces contractile muscle activity. When the pulse duration was extended to 50 msec, the level of secreted IL-6 in the media from contracted myotubes increased compared to basal media without stimulation. However, β-actin levels also increased in the media, indicating that the increase in IL-6 levels in the conditioned media correlated with cell damage (Fig 2B).

### Medium exchange-induced protein secretion from C2C12 myotubes

**Fig 3:**
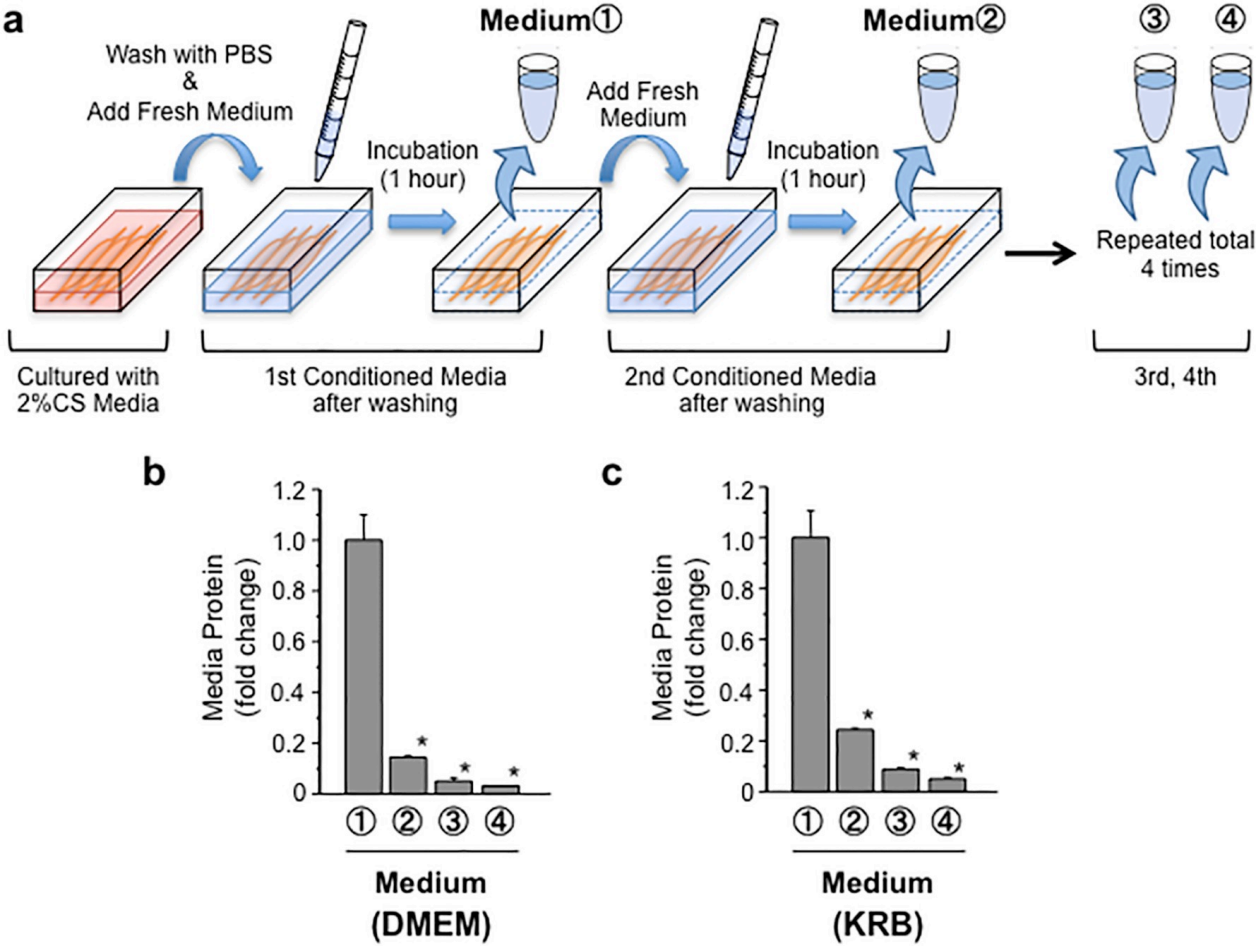
Medium exchange induces the secretion of large amounts of protein from C2C12 myotubes. **(A) Experimental scheme**. C2C12 myotubes were washed twice with PBS and then fresh buffer was added. After a 1-hour incubation, the conditioned medium was collected as the 1st medium marked with “①”. Next, fresh buffer was again added to the myotubes and collected as the 2nd medium (“②”) after an additional 1-hour incubation. This procedure was repeated four times. **(B) Time-dependent changes in protein levels in the conditioned media from C2C12 myotubes**. Total protein levels in conditioned media were determined every hour. For this experiment, both DMEM and KRB buffers were used. Values are expressed relative to the protein levels detected in the medium after the first hour and are presented as the mean ± SEM (n = 4). *: *p* < 0.05 compared with the first hour.

The culture medium must be replaced with fresh medium at the beginning of the myotube incubation to quantify the secreted myokine concentrations. During a series of preliminary experiments, we fortuitously noticed that the exchange of culture medium provoked the unexpected release of proteins from the myotubes. The medium exchange and the 1-hour incubation of myotubes were repeated four times in a continuous manner, as shown in Fig 3A. The highest protein concentrations, which were quantified using the Bradford method, were detected in the first fraction of conditioned media compared to those in subsequent fractions (Fig 3B). We also performed the same experiment using Krebs-Ringer Bicarbonate (KRB) buffer and obtained a similar result (Fig 3C). The high levels of protein in the first collected fraction were not calf serum proteins contained in the different types of medium because the electrophoretic protein patterns were entirely different between these samples (S1A Fig). These data suggested a new biological feature of skeletal muscle cells, specifically that large amounts of protein are released in response to medium exchange.

Since changes in osmotic pressure might produce stress responses in myotubes, the osmolality of the different types of medium, including 2% calf serum, phosphate buffered saline (PBS), serum-free DMEM, and KRB buffer, was evaluated. However, significant differences in the osmotic pressure were not observed among these solutions (S1B Fig). We also washed the myotubes with DMEM rather than PBS and observed the same pattern of protein release (S1C Fig). Interestingly, the medium exchange-induced protein release was specifically observed in differentiated myotubes. Lower protein concentrations were detected in the medium of C2C12 myoblasts, which are undifferentiated muscle cells, compared to those in the medium of C2C12 myotubes. In addition, protein release was reduced in non-muscle cells, such as Chinese hamster ovary cells (S1C Fig).

Because protein secretion from secretory cells is regulated by an increased of intracellular calcium ion concentration ([Ca^2+^]), we examined the effect of the medium change on Ca^2+^ flux in C2C12 myotubes using the Fluo-8 dye solution (AAT Bioquest Inc., Sunnyvale, CA, USA). As shown in S1D Fig, the fluorescence of Ca^2+^ was enhanced immediately after adding fresh medium, suggesting the possibility that calcium signaling is involved in the medium change-induced protein release. Currently, we do not have an explanation for this phenomenon, including the detailed mechanism triggering protein secretion and the identities of the released proteins and the specific stimulus that induces release. However, these data raise the possibility that this abundant protein release process might mask contraction-induced IL-6 secretion.

### Elimination of the protein release to unmask contraction-induced IL-6 secretion in C2C12 myotubes

**Fig 4:**
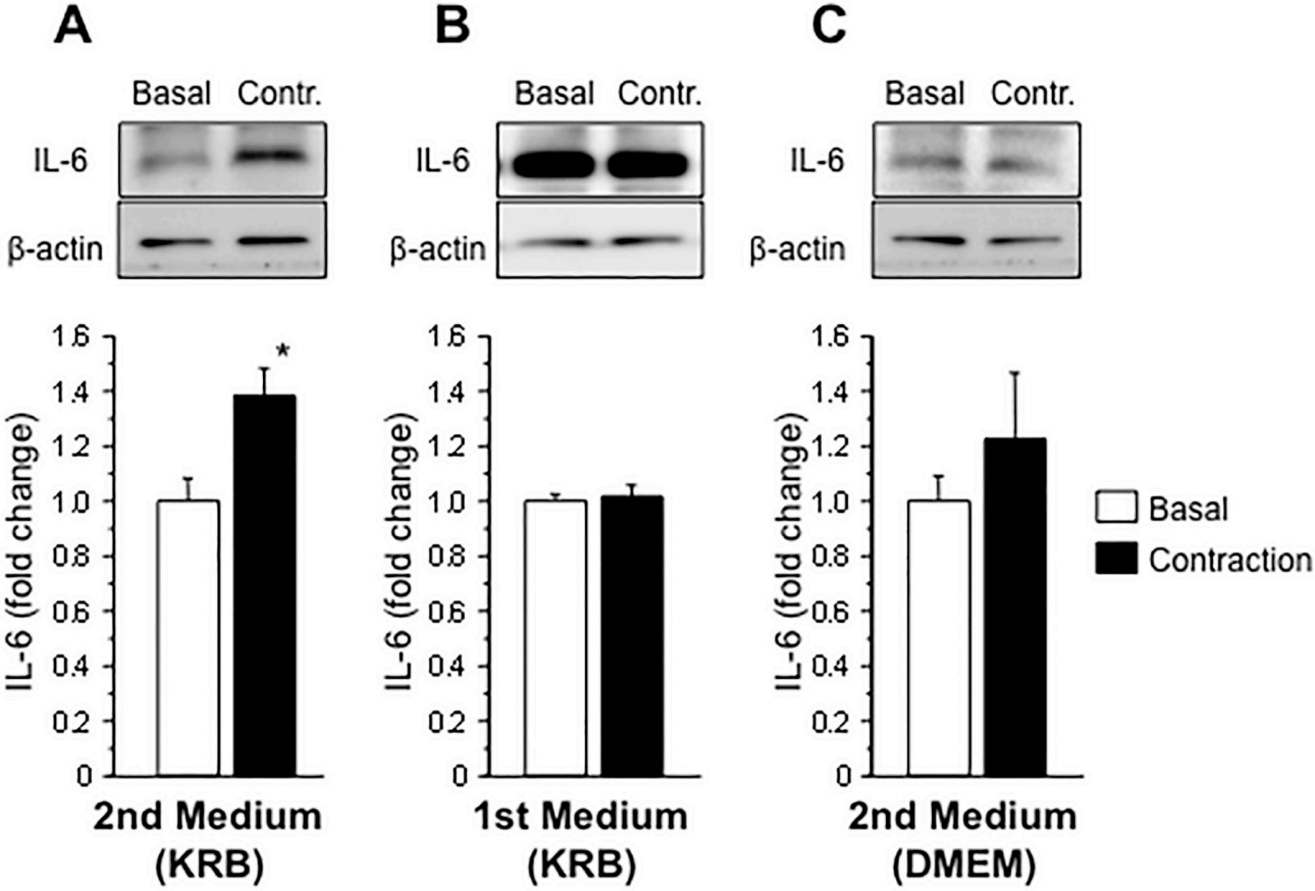
Determination of the experimental conditions for contraction-induced IL-6 secretion from C2C12 myotubes. The concentrations of IL-6 and β-actin secreted by C2C12 myotubes into the conditioned medium were determined in once- or twice-exchanged KRB buffer or DMEM. IL-6 secretion increased following muscle contraction after the buffer was exchanged twice with KRB buffer. However, no significant changes in IL-6 secretion were observed between the other experimental conditions. Values are expressed relative to the amount in basal medium and are presented as the mean ± SEM (n = 4–5). *: *p* < 0.05 compared with basal medium.

Since protein release was substantially diminished in the second conditioned medium (Fig 3), we expected that the IL-6 secretion evoked by muscle contraction might be observed in the second conditioned medium. The first conditioned medium, including miscellaneous proteins, was discarded, and myotubes were then contracted by electrical stimulation for 1 hour in the second medium. The electrical current and pulse duration for the stimulation were set to 15 mA and 20 msec, respectively. As expected, IL-6 secretion from myotubes was detected in the second conditioned medium after contraction in the absence of cellular damage (Fig 4A). Similar to previous results, an increase in IL-6 secretion was not observed in the first conditioned medium in a contraction experiment performed simultaneously (Fig 4B). Thus, contraction-induced IL-6 secretion is masked by protein release induced by buffer exchange. In addition, the KRB buffer, rather than DMEM, is better for detecting IL-6 secretion (Fig 4C). Moreover, the intracellular IL-6 levels in C2C12 myotubes were not altered by muscle contraction (S2A Fig), indicating that the increase in IL-6 levels in the culture medium was not a consequence of the increased *de novo* synthesis following contraction but was induced by a mechanism regulating secretion. To investigate whether IL-6 secretion from C2C12 myotubes is mediated by exosomes, we extracted exosomal vesicles from conditioned media and examined the presence of IL-6 proteins in the exosome fraction. We confirmed that CD9, an exosome-specific protein marker, was present in the exosome fraction, while IL-6 was not detected in this fraction. Based on these data, IL-6 secretion is not mediated by exosomes (S2B Fig).

### The trigger of contraction-induced IL-6 secretion

**Fig 5:**
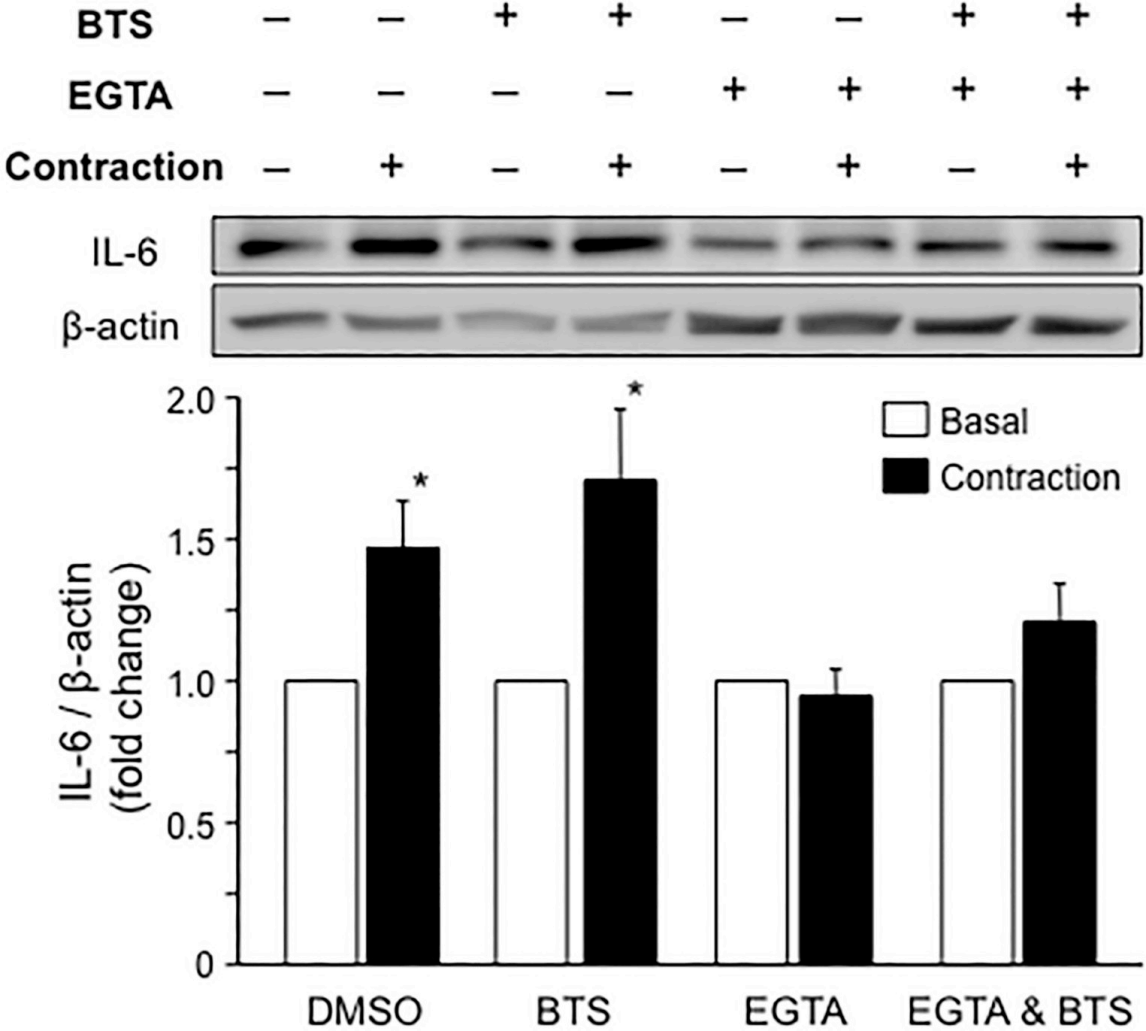
Effects of BTS and EGTA on contraction-induced IL-6 secretion by C2C12 myotubes. C2C12 myotubes treated with BTS (50 μM) and/or EGTA (10 mM) were electrically contracted for 1 hour. Levels of the IL-6 protein in the conditioned media were quantified by western blotting. IL-6 secretion increased following electrical stimulation in the presence of DMSO and BTS but not in the presence of EGTA. Values are expressed relative to the basal medium and are presented as the mean ± SEM (n = 15). *: *p* < 0.05 using ANOVA with Dunnett’s post hoc test.

We used pharmacological inhibitors of muscle contraction and examined whether contraction-induced IL-6 secretion disappeared following treatment with these inhibitors to identify the specific mechanism regulating contraction-induced IL-6 secretion in skeletal muscle cells. BTS is a specific inhibitor of myosin ATPase that blocks the cross bridge cycling of actin and myosin without affecting Ca^2+^ flux from the sarcoplasmic reticulum [27, 28]. As expected, the blockade of myosin ATPase with BTS completely inhibited muscle contractile movements, but the Ca^2+^ flux evoked by electrical stimulation was fluorescently detected using the Fluo-8 dye (S1 File). IL-6 secretion also occurred in response to the BTS treatment following electrical stimulation (Fig 5), indicating that the mechanosensory transduction generated by muscle contraction is not involved in the mechanism of acute IL-6 secretion. We next used the calcium-chelating agent EGTA to examine the effect of inhibition of Ca^2+^ flux. In this experiment, we confirmed that both the fluorescent Ca^2+^ flux and muscle contractile movement disappeared following an electrical pulse (S1 File). Interestingly, contraction-induced IL-6 secretion was completely abolished in the presence of EGTA. Furthermore, contraction-induced IL-6 secretion was also blocked in the presence of both BTS and EGTA. Thus, calcium flux, rather than contraction itself, triggers contraction-induced IL-6 secretion.

### Confirmation of regulated myokine secretion using the newly determined experimental conditions

**Fig 6:**
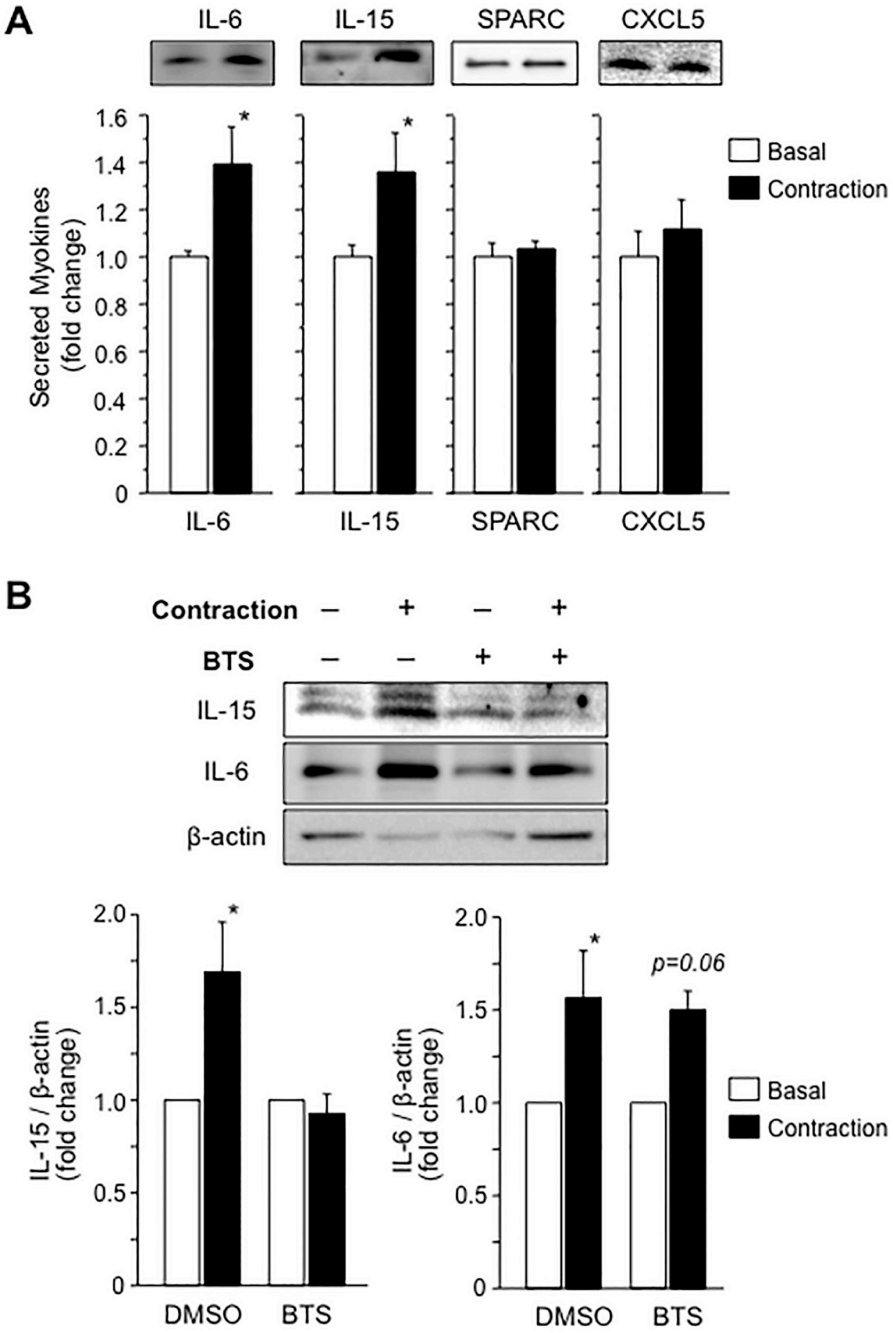
Regulation of myokine secretion in response to a 1-hour muscle contraction under the newly established experimental condition. **(A)** The levels of previously reported myokines in conditioned media from myotubes treated with or without electrical stimulation were detected and quantified by western blotting. IL-6 and IL-15 secretion increased following a 1-hour muscle contraction. No significant changes in SPARC and CXCL5 levels were observed between C2C12 myotubes cultured under basal conditions and contracted C2C12 myotubes. Values are expressed relative to the basal medium and are presented as the mean ± SEM (n = 5–11). *: *p* < 0.05 compared with the basal medium. **(B)** Effect of BTS on contraction-induced IL-6 and IL-15 secretion. IL-15 secretion was not increased by muscle contraction in the presence of BTS. Values are expressed relative to the basal medium and are presented as the mean ± SEM (n = 5). *: *p* < 0.05 using the Kramer post hoc test.

Since we established the experimental conditions for secretion induced by acute contraction, we investigated whether the secretion of previously reported myokines was regulated by muscle contraction. IL-15 has been described as a contraction-induced myokine because its plasma level increases in humans during running [29]. Although IL-15 has not been shown to be secreted from skeletal muscles in cultured cells, IL-15 secretion by C2C12 myotubes increased in response to acute contraction in the present study (Fig 6A). These results support findings from a previous study showing that moderate intensity treadmill running for 30 minutes significantly increases circulating IL-15 levels in humans [29]. We examined the effect of BTS on IL-15 secretion. Although IL-6 secretion was again significantly increased following electrical stimulation, even in response to BTS, as shown in Fig 6B, IL-15 secretion was surprisingly completely abolished under the same condition (in the same medium sample) (Fig 6B). Based on these data, IL-15 secretion is regulated by a different mechanism than IL-6 secretion.

Secreted protein acidic and rich in cysteine (SPARC) is also considered a myokine regulated by muscle contraction since the plasma SPARC level increases following a single bout of exercise [30]. Direct evidence for SPARC secretion by muscle cells was provided by an experiment using the cyclic stretching experiment, despite electrical stimulation. However, we did not observe an increase in SPARC secretion following electrical stimulation (Fig 6A). We also examined the contraction-induced regulation of CXCL5 secretion because this protein was reported to be secreted following muscle contraction for 24 hours [14]. However, using our protocol, CXCL5 levels in conditioned media were not altered between the contracted and basal C2C12 myotubes (Fig 6A).

## Discussion

Contraction-induced myokines are especially of scientific interest and clinically attractive, because these molecules may potentially account for exercise-induced health benefits, and may be used in drug discovery. However, direct evidence for myokine secretion induced by acute contraction was lacking. Importantly, we noticed that exchange in the cell culture medium unexpectedly triggered a substantial release of proteins from C2C12 myotubes, which obscured the contraction-induced myokine secretion. Once these proteins were eliminated, IL-6 exhibited increased secretion in response to muscle cell contraction induced by electrical stimulation, in the absence of cellular damage.

The reported increase in the level of IL-6 in human plasma after acute exercise is consistent across numerous studies [31]. However, the up-regulation of IL-6 was thought to be the consequence of an immune response, or due to leakage from damaged muscle cells, rather than protein secretion by intact muscle cells. This is because the kinetics of plasma IL-6 are associated with the concentration of creatine kinase, a muscle damage indicator [32]. On the other hand, some groups argued that the increase in IL-6 in the plasma in response to exercise is independent of muscle damage, and implied the existence of another mechanism, by which exercise can induce the increase in plasma IL-6 [31]. In the present study, the intensity of contraction was set lower than the level at which muscle damage is induced to clearly distinguish myokine secretion from leakage. Thus, we directly demonstrated that the secretion of IL-6 increased following muscle contraction, in the absence of cellular damage, suggesting the presence of secretory machinery in skeletal muscle cells.

We demonstrated that the calcium flux is important for regulating IL-6 secretion in inhibitory experiments. The involvement of calcium signaling in IL-6 secretion by skeletal muscle cells is consistent with the hypothesis that continuous muscle contraction induces IL-6 transcription through calcium signaling [17]. Researchers have shown that IL-6 transcription is induced by nuclear factor of activated T-cell (NFAT) through the action of calcineurin, a serine/threonine phosphatase, which is activated by a sustained elevation in the concentration of intracellular Ca^2+^ [33]. Since muscle contraction represents a potent stimulus for the release of Ca^2+^ from the sarcoplasmic reticulum, it is generally understood that continuous muscle contractile activity increases IL-6 expression through the Ca^2+^/NFAT pathway [34]. However, the IL-6 secretion induced by acute contraction was not accompanied by the upregulation of the expression of intracellular IL-6. Therefore, another pathway, different from NFAT signaling, must be responsible for the observed results.

Using our model, we have determined whether a myokine is clearly secreted in response to muscle contraction. Specifically, IL-15 is also secreted by acute muscle contraction. These results support a previous study, which had reported that moderate intensity treadmill running for 30 minutes resulted in a significant increase in circulating IL-15 levels in humans [29]. Since the muscle specific overexpression of IL-15 can reduce the fat mass [35], muscle derived IL-15 is an important mediator for regulating body composition. Interestingly, IL-15 secretion was inhibited by BTS, indicating that the mechanism of IL-15 secretion is different from that of IL-6 secretion. Our established experimental condition is suitable not only for the discovery of novel contraction-induced myokines, but also for the dissection of the regulatory mechanism underlying myokine secretion.

IL-6 has been described as a proinflammatory cytokine that contributes to the development of insulin resistance in skeletal muscle [36]. IL-6 levels are chronically elevated in obese humans (~2- to 3-fold) and appear to be linked to chronic low-grade inflammation [37]. However, exercise-induced IL-6 secretion has a beneficial role in glucose metabolism in skeletal muscle because IL-6-deficient mice show decreased glucose uptake induced by acute contraction and decreased exercise training-induced insulin sensitivity [38]. One important reason for the paradoxical effects of IL-6 on glucose metabolism is the duration of IL-6 secretion. Although the disease-associated elevation in IL-6 levels is persistent, the exercise-induced elevation of plasma IL-6 concentrations is transient (only during exercise) [39]. In fact, according to Nieto-Vazquez et al., a short-term IL-6 treatment activated glucose uptake in an additive manner with insulin, resulting in improved glucose tolerance and insulin sensitivity, whereas chronic exposure resulted in insulin resistance [40]. Although several studies have implied that exercise results in an acute and transient increase in IL-6 levels, our study is the first to reveal that the exercise-associated elevation in IL-6 levels is derived from contracting skeletal muscle cells.

Importantly, C2C12 myotubes release large amounts of proteins after buffer exchange to serum free conditioned medium, and these proteins mask the contraction-regulated IL-6 secretion. Interestingly, protein flooding does not occur in non-muscle cells or undifferentiated muscle cells; thus, this is a muscle-specific phenomenon. Although the mechanism underlying the buffer exchange-induced protein release was unclear (although, at least osmolality was eliminated as a cause), skeletal muscle has the ability to secrete a wide variety of proteins, in response to some types of stimulation. Considering that skeletal muscle is the largest organ, comprehensive investigations are needed to understand the endocrinology of skeletal muscles.

## Conclusion

We concluded that acute muscle contraction apparently promotes the secretion of some myokines, and that regulatory secretion system of myokines exits in skeletal muscle cells. Our newly established experimental model will enable researchers to adopt a proteomic approach to identify new myokines secreted in response to muscle contraction. The identification of new myokines and an understanding of their regulatory mechanisms will be useful in the development of medicines.

## Supporting information

S1 Fig (A) Coomassie blue-stained SDS-PAGE gel of calf serum and the conditioned medium after medium exchange. The left lane contains a molecular marker, which indicates the molecular weight of the bands. (B) Osmotic pressure was compared between the differentiation medium (DMEM supplemented with 2% calf serum), PBS, serum-free DMEM, and KRB buffer. (C) Comparison of the secreted protein levels between the first and second media, after buffer exchange, and different conditions or cell lines. Myotubes were washed with PBS and serum-free DMEM. Myoblasts and CHO cells were washed with PBS. Conditioned media samples were collected, and the levels of secreted proteins were quantified. (D) C2C12 myotubes were incubated with Fluo-8 and [Ca^2+^] flux was visualized. After addition of fresh medium, the fluorescence of calcium was enhanced compared to the basal state.
(TIFF)

S2 Fig (A) Intracellular levels of the IL-6 protein were compared between myotubes maintained in a basal state and C2C12 myotubes contracted for 1 hour. The quantitative data are presented as a ratio relative to the β-actin level. (B) Exosomal vesicles from conditioned media of C2C12 myotubes were isolated using ExoQuick-TC ULTRA EV Isolation Kit (System Biosciences, Palo Alto, CA). Exosome-specific marker CD9 was detected in the exosome fraction, but IL-6 was not detected in the same fraction. Control is a whole conditioned medium, which is not fractionated, obtained by a methods described in the Method.
(TIFF)

S1 File Effects of muscle contraction inhibitors on the muscle contractile movement and [Ca^2+^] flux evoked by electrical stimulation. C2C12 myotubes were treated with BTS (50 mM) and EGTA (10 mM) and were incubated with Fluo-8, a calcium indicator. C2C12 myotubes were observed under a phase-contrast microscope, and fluorescence was determined in the same area. The fluorescent [Ca^2+^] flux was observed in DMSO- or BTS-treated myotubes following the administration of an electric pulse, but the fluorescent signal disappeared following the EGTA treatment. Fluo-8 dye solution was used at a 25% dilution relative to that recommended by the manufacturer for calcium imaging.
(MP4)
