# Supplementary figures and images for: Evidence for acute contraction-induced myokine secretion by C2C12 myotubes
Source: PLoS One. 2018 Oct 24;13(10):e0206146. doi: 10.1371/journal.pone.0206146 (PMC6200277; doi:10.1371/journal.pone.0206146)

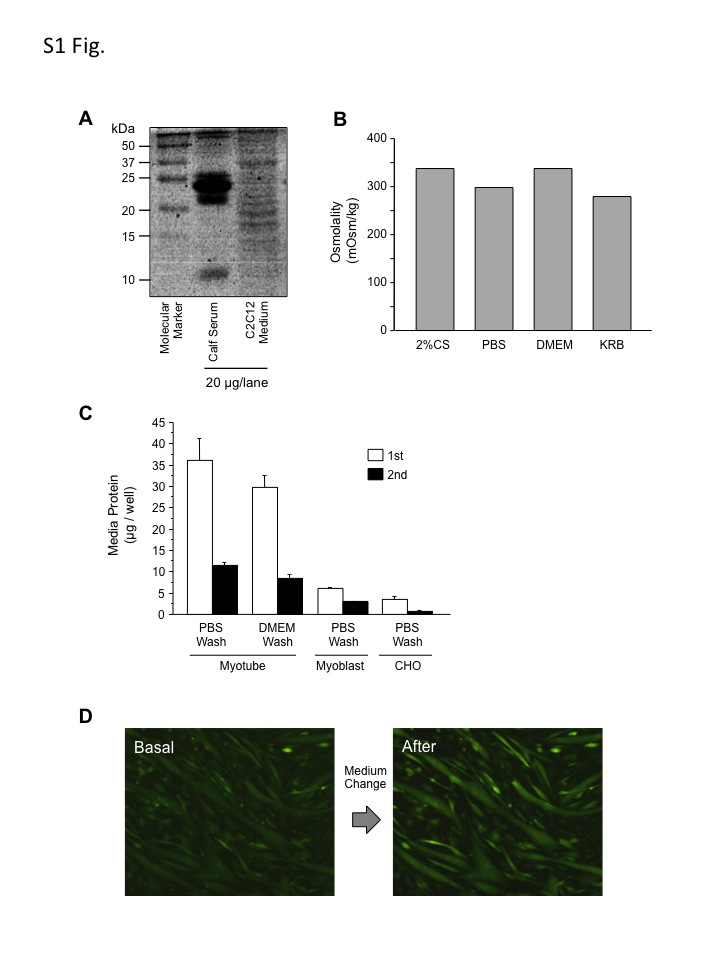

Supplement: S1 Fig — (A) Coomassie blue-stained SDS-PAGE gel of calf serum and the conditioned medium after medium exchange. The left lane contains a molecular marker, which indicates the molecular weight of the bands. (B) Osmotic pressure was compared between the differentiation medium (DMEM supplemented with 2% calf serum), PBS, serum-free DMEM, and KRB buffer. (C) Comparison of the secreted protein levels between the first and second media, after buffer exchange, and different conditions or cell lines. Myotubes were washed with PBS and serum-free DMEM. Myoblasts and CHO cells were washed with PBS. Conditioned media samples were collected, and the levels of secreted proteins were quantified. (D) C2C12 myotubes were incubated with Fluo-8 and [Ca2+] flux was visualized. After addition of fresh medium, the fluorescence of calcium was enhanced compared to the basal state. (TIFF) [file pone.0206146.s001.tiff]

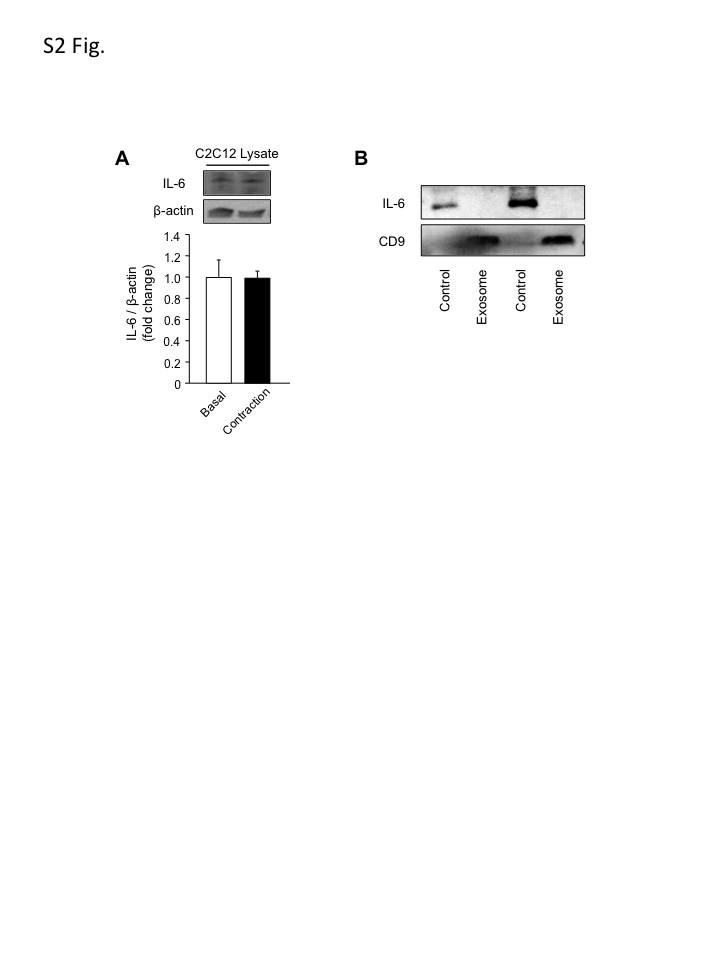

Supplement: S2 Fig — (A) Intracellular levels of the IL-6 protein were compared between myotubes maintained in a basal state and C2C12 myotubes contracted for 1 hour. The quantitative data are presented as a ratio relative to the β-actin level. (B) Exosomal vesicles from conditioned media of C2C12 myotubes were isolated using ExoQuick-TC ULTRA EV Isolation Kit (System Biosciences, Palo Alto, CA). Exosome-specific marker CD9 was detected in the exosome fraction, but IL-6 was not detected in the same fraction. Control is a whole conditioned medium, which is not fractionated, obtained by a methods described in the Method. (TIFF) [file pone.0206146.s002.tiff]
